# Information-seeking behavior on sexually transmitted infections and its associated factors among university students in Ethiopia: a cross-sectional study

**DOI:** 10.1186/s12978-022-01340-x

**Published:** 2022-01-29

**Authors:** Aynadis Worku Shimie, Kassahun Dessie Gashu, Atsede Mazengia Shiferaw, Shegaw Anagaw Mengiste

**Affiliations:** 1 Department of Health Informatics, Institute of Public Health, College of Medicine and Health Sciences, University of Gondar, P.O. Box 196, Gondar, Ethiopia; 2 School of Business, Institute of Business, History & Social Sciences, University of South-Eastern Norway, Notodden, Norway

**Keywords:** STIs, STIs Information seeking behavior, University students, Sexual health information, Ethiopia

## Abstract

**Background:**

Sexually Transmitted Infections (STIs) are infections commonly spread through sexual contact and transmitted by bacteria, viruses, or parasites. In today’s world, STI-related information-seeking behavior is often vital for the advancement of knowledge, behavioral changes, health decisions, and the sharing of sexual health information among youths. However, paucity of evidence on information-seeking behavior among students in higher education institutions. This study aimed to assess STI-related information-seeking behavior and its associated factors among students at the University of Gondar.

**Methods:**

An institution-based cross-sectional quantitative study was conducted among students at the University of Gondar from January 15 to February 15, 2021. A total of 832 participants were selected using a stratified two-stage sampling method. A structured self-administered questionnaire was used to collect the required data. STI information-seeking behavior questionnaire was adapted from health information national survey tool (HINTS). Descriptive statistics, bi-variable, and multivariable logistic regression analyses were applied using SPSS version 26.

**Result:**

The proportion of STI related information-seeking among university students was 462 (55.5%) with 95% CI (52.3, 58.9). About 263 (56.9%) of students preferred internet as a primary source for STI related information. Year of study being 4th (AOR = 4.77, 95% CI = 2.75, 8.29) and 5th year (AOR = 5.45, 95% CI = 2.48, 12.01), field of study being health (AOR = 2.19, 95% CI = 1.16, 4.11), sexual experiences (AOR = 2.33, 95% CI = 1.56, 3.48), ever had STI symptoms (AOR = 4.19, 95% CI = 2.14, 8.18), perceived susceptibility (AOR = 5.05, 95% CI = 3.29, 7.75), and perceived severity (AOR = 2.16,95% CI = 1.45, 3.22) were significant factors for good STI information-seeking.

**Conclusion:**

the proportion of STI information-seeking among university students was low. Students' STI information-seeking behavior could be improved by increasing digital literacy and enhancing computer and internet access across the campus.

## Plain language summary

Sexually Transmitted Infections (STIs) are infections commonly spread through sexual contact and transmitted by bacteria, viruses, or parasites and are the most common cause of illness among university students. Information-seeking behavior related to STIs is a means by which individuals sought information related to sexual health, disease, health risk, and health promotion. Communicating STIs and sexual risk behaviors during youth, particularly among university students, is effective in reducing STI-related problems. Lack of access to health information, lack of communication, and insufficient information about STIs are key issues that have contributed to the high prevalence of STIs. In Ethiopia, information-seeking behavior related to STIs and information use culture for improving the sexual health of individuals and the communities is limited. Therefore, the purpose of this study was to assess STI information-seeking behavior and its associated factors among students at the University of Gondar.

More than half of the students sought STI-related information seeking in the past year. From those STI-related information seekers, the majority of students preferred the internet as a primary source for STI-related information. Field of study, year of study, internet access, digital literacy, sexual experiences, had STI symptoms, Perceived susceptibility, and perceived severity of STI were significant factors associated with STI information-seeking. Students' STI information-seeking behavior could be improved by increasing digital literacy and enhancing computer and internet access.

## Introduction

Sexually Transmitted Infections (STIs) are infections commonly spread through sexual contact and transmitted by bacteria, viruses, or parasites. STIs have a significant influence on sexual and reproductive health around the world [1]. According to a World Health Organization (WHO) report in 2018, sexually transmitted infections (STIs) have become a major global health issue, with more than one million individuals are suffering from curable sexually transmitted infections per day [2].

In Southeast Asian countries like Malaysia, the proportion of health information-seeking among university students was 80% [3]. Evidence showed that sub-Saharan Africa was the region with the highest STI category and it has a high public health problem. [4] Another study showed that students' level of STI information-seeking in Nigeria was 73.6% [5]. Various studies have shown that to prevent STIs and to improve sexual health in universities, educators, health experts, and university administrative staff should focus on increasing sexual health literacy and promoting health information-seeking abilities [6]. Although universal access to information is a requirement for achieving Health for All [7], lack of access to health information remains a major barrier to knowledge-based health care in developing countries. This works for the majority of Ethiopian students who do not have access to credible, relevant, and useable information about STIs. In Ethiopia, STIs have been reported as a problem among university students [8].

According to data from the Ethiopian Federal Ministry of Health report, those aged 15–24 have the highest reported incidence of STIs [9]. In Ethiopia, the majority of university students are in this age group. A study conducted in Ethiopia on students at the University of Gondar showed that STI prevalence among students was 18.2% [10].

STI information-seeking behavior is a means by which individuals sought information related to health, disease, health risk, and health promotion [11]. As a result, Individuals can better grasp their STI concerns and information needs. People seek information on sexually transmitted infections first, analyze it, and then use it to meet their information needs [12]. Communicating STIs and sexual risk behaviors during youth, particularly among university students, is effective in reducing STI-related problems. In all health systems, quality and reliable health information provide a foundation for providing improved health services and enhancing informed decision-making [13]. It helps to advance and implement healthcare policy, governance, and regulation, as well as health-related research, human resource development, health education and training, service delivery, and healthcare funding [14]. Furthermore, because the information is critical for every individual, access to and use of the Electronic Information System for clinical decisions is critical to achieving health-related sustainable development goals [15].

In developing countries like Ethiopia, information-seeking behavior and information use culture for improving the health of individuals and the communities is limited [16]. Furthermore, lack of access to health information and information-seeking behavior, lack of communication, and insufficient information about STIs are key issues that have contributed to the high prevalence of STIs [17]. The study believes it is essential for students to have access to health information about their sexual and reproductive health, as well as information on sexual risk behaviors, through their preferred information sources [18]. Because university students are sexually active members of society, it is necessary to investigate their sexual health information environment. This is because many students join the University with little information about sexual health, and as a result, they regularly participate in sexual activities that put them at bad health outcomes such as HIV and other STIs [19]. Therefore, it is vital to encourage students to obtain sexual health information to improve their sexual health, promote safer sex habits, and reduce the transmission of STIs and HIV. In today's world, health information is frequently used for knowledge advancement, behavioral changes, health decision-making, and the sharing of health-related information [20]. Therefore, the purpose of this study was to assess STI information-seeking behavior and its associated factors among University of Gondar students.

## Methods

### Study design and setting

An institution-based cross-sectional study was undertaken among regular undergraduate students at the University of Gondar from January 15 to February 15, 2021. The University of Gondar is one of Ethiopia's oldest universities located in the historic town of Gondar. The University began as a Public Health College and Training Center in 1954 [21]. The University currently hosted more than 40,000 students who came from different parts of the country. Last year, the university enrolled a total of 19,646 undergraduate students [21]. To increase awareness of adolescent and reproductive health issues, the university has established HIV clubs and resource centers.

### Study population

This study included all regular undergraduate students from selected departments at the University of Gondar as participants. Regular undergraduate students from selected departments at the University of Gondar who were available during the data collection period and were in their second year or higher were included in this study.

### Sample size and sampling procedures

The sample size was estimated using single population proportion formula with assumptions of 95 percent confidence interval (CI), Z (1−α/2) = 1.96), a 50% predicted proportion of STI Information-seeking behavior (p), and a 5% margin of error (d). A total of 844 students were used as a sample, with a design effect of two and a 10% non-response rate. A stratified two-stage sampling approach was used to select study participants. In the first stage, departments were divided into two categories: health-related and non-health-related disciplines, to deal with the fact that STI information-seeking varies by field of study, and then proportional allocation was employed to select the study population using simple random sampling. In the second stage, students were stratified by year of study, with the number of students in each year of the selected department proportionally divided, taking into account that their years of study and length of stay at the University can affect STI information-seeking behavior. Each study participant was selected using a simple random sampling approach.

### Data collection tools and procedures

A pre-tested and structured self-administered questionnaire was used to collect data from study participants. The study's instrument (tool) was adapted from other research and modified to satisfy our context and objectives. The health information national survey tool (HINTS) [22] was used to adapt the STI information-seeking behavior questionnaire, and the psychological components tool was adapted from the health belief model (HBM) [23]. Different questions measuring socio-demographic characteristics, STI information sources, behavioral characteristics, psychological factors, technological factors, and health-related factors were included in the data collection instrument. Data collectors and supervisors were trained about the data collection procedures. The Data were collected by 5 data collectors supervised by two supervisors. Pretest for the questionnaire was made on about 10% of the total studied population at Debre Markos University which is outside of the study area. Before the real data collection began, some misunderstandings and errors were corrected. After data collection, questionnaires were reviewed and checked for completeness, and the data was cleaned to check for errors and missing information.

### Measurements

STI information Seeking was measured with one item question derived from the previous study that determines whether respondents sought STI information in the past 12 months. [15] Subsequently, the participants were asked about the frequency of STI information seeking. STI information-seeking behavior can be characterized by the STI information sources, types of STI information, trust in STI information sources, and reasons for seeking STI information [3, 24].

### Data processing and analysis

The data entry form was created using Epi-Info 7.2, and the analysis was done using SPSS version 26. Factors associated with STI information-seeking were assessed using bi-variable and multivariable logistic regression. The model fitness was checked using Hosmer and Leme show tests, with a p-value of 0.593. In bi-variable logistic regression, variables with p-value < 0.2 was considered for multivariable logistic regressions. Before conducting a multivariable logistic regression, multicollinearity was done. In multivariable logistic regression, the variables with a P-value of less than or equal to 0.05 were considered significant. The results were presented in the form of tables, figures, and text, with frequencies and summary statistics such as mean, standard deviation, and percentage used to summarize the study population concerning relevant variables.

### Ethics considerations

Ethical clearance was received from the Institutional Review Board (IRB) of the University of Gondar CMHS institute of public health with reference No /IPH/1314/2013. Oral consent was obtained from each study participant after they were told of the study's purpose and objective. To ensure the confidentiality of any information provided by study participants, the data collection method was secret and the dataset did not include personal identifiers.

## Result

### Sociodemographic characteristics

A total of 832 study subjects participated in this study with a 98.6% response rate. The mean age of the study participants was 23.09 ± 1.85 SD years with ranges from 20 to 36 years, and 483 (58.1%) of the respondents were male. The result indicated that around 466 (56%) of the study participants have previously resided in rural. The majority, 568 (68.3%) of the study participants have not participated in any voluntary health club, and 736 (88.5%) of the study participants were non-health students. Regarding religion, around 626 (75.2%) of the study participants were Orthodox, 78 (9.4%) of the study participants were Muslim, 108 (13.0%) of the study participants were Protestant, and 20 (2.4%) of the students were Catholic. The result showed that the marital status of 767 (92.2%) of study participants was single and around 51 (6.1%) of the study participants were married (Table 1).

**Table 1:** Sociodemographic characteristics of Respondents at University of Gondar, Northwest Ethiopia, 2021 (n = 832)

Variables	Category	Frequency	Percent
Age (in years)	< 23	489	58.8
	> 23	343	41.2
Gender	Male	483	58.1
	Female	349	41.9
Prior residence	Urban	366	40.0
	Rural	466	56.0
Health Club participation	Yes	264	31.7
	No	568	68.3
Religion	Orthodox	626	75.2
	Muslim	78	9.4
	Protestant	108	13.0
	Catholic	20	2.4
Field of study	Health-related	96	11.5
	Non-health-related	736	88.5
Year of study	2nd year	241	29.0
	3rd year	335	40.3
	4th Year	180	21.6
	5th Year	76	9.1
Marital status	Single	767	92.2
	Married	51	6.1
	*Others	14	1.7

Others* contains separated, widowed, and divorced

### Psychological characteristics

Of the total respondents, about 307 (36.9%) perceived that they were very concerned about STI, and less than half 335 (40.3%) perceived that STI is a severe disease. More than half of the respondents 714 (85.8%) had high confidence about their ability to take good care of their health (*Table **2*).

**Table 2:** Psychological factors to STI information-seeking behavior among university students

Variables	Categories	Frequency	Percent (%)
Perceived Susceptibility to STI	Concerned	307	36.9
	Not concerned	525	63.1
Health self-efficacy	Confident Not confident	714 118	85.8 14.2
Perceived severity of STIs	Severe	335	40.3
	Not severe	497	59.7

### Technological characteristics

The majority of the respondents 647 (77.8%) and 723 (86.9%) had computer access and internet access at their campuses respectively. Of the total participants, about 371 (44.6%) respondents had high digital literacy (Table 3).

**Table 3:** Technological factors to STI information-seeking behavior among students at University of Gondar, 2021

Variables	Category	Frequency	Percentage (%)
Digital literacy	High	371	44.6
	Low	461	55.4
Computer access	Yes No	647 185	77.8 22.2
Internet access	Yes No	723 109	86.9 13.1

### Behavioral and Health-related characteristics

About 69 (8.3%) of the study participants had a history of alcohol drinking and around 70 (8.4%) had a history of cigarette smoking. Around 293 (35.2%) of the study participants had a history of sexual experience. From the study participants who had sexual experiences, 167 (57.2%) were used a condom during sexual intercourse. Regarding current health conditions, about 756 (90.9%) of the students feel healthy and 85 (10.2%) of the participants showed STI signs and symptoms. From all study participants, about 284 (34.1%) of students were tested for STI in the last 12 months, only 36 (4.3%) of the participants had a personal history of STI, and 87 (11.4%) of the participants had a family history of STI (*Table **4*).

**Table 4:** behavioral and health-related factors to STI information-seeking behavior among university students

Variables	Category	Frequency	Percentage
Alcohol drinking	Yes	69	8.3
	No	763	91.7
Smoking	Yes No	70 762	8.4 91.6
Sexual experience	Yes No	293 539	35.2 64.8
Perceived health status	Feel healthy Feel less healthy	756 76	90.9 9.1
Had STI symptoms	Yes No	95 737	11.4 88.6
Had STI test	Yes No	284 548	34.1 65.9
STI history	Yes No	36 796	4.3 95.7
Family STI history	Yes Not sure No	87 173 572	10.5 20.7 68.8

### Information-seeking behavior on sexually transmitted infections

**Fig. 1:**
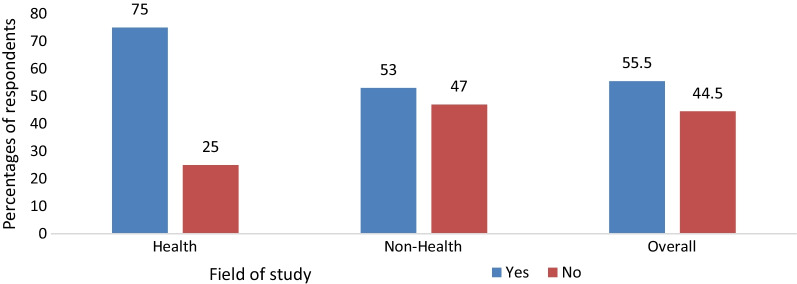
STI information seeking by students’ field of study in University of Gondar, 2021

From total respondents, more than half 462 (55.5%) of participants had STI information-seeking behavior in the past 12 months. From the total STI information seekers, about 300 (64.9%) were males, and 72 (75%) of health science students have sought STI information (Fig. 1).

### Frequency of STI Information seeking

**Fig. 2:**
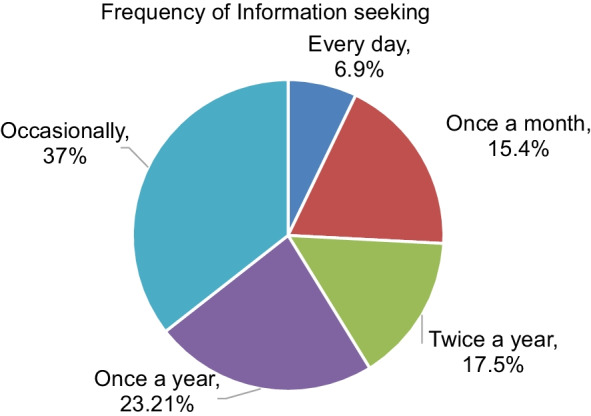
Frequency of STI information seeking by students in the university of Gondar 2021 (n = 462)

Regarding the frequency, about 171 (37%) of STI information seekers sought information occasionally, about 107 (23.21%) of them sought once a year, and 81 (17.5%) of them sought it twice a year (Fig. 2).

### Source of health information

**Fig. 3:**
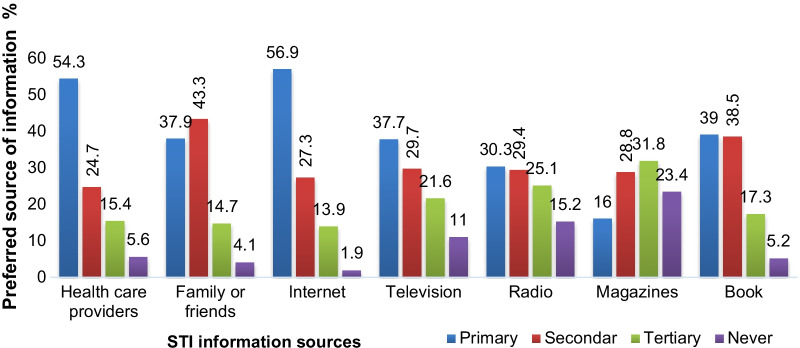
Preferred STI information source of students in the University of Gondar, 2021 (n = 462)

Participants were asked about the source of STI information. The result of this study indicated that 263 (56.9%) of STI information seekers preferred the internet as their primary STI information sources followed by health care providers 251 (54.3%). In contrast, magazine 74 (16%) and radio 140 (30.3%) were the least preferred STI information sources. Other STI information sources used by students were books, family or friends, and televisions (Fig. 3).

### Type of STI Information

**Fig. 4:**
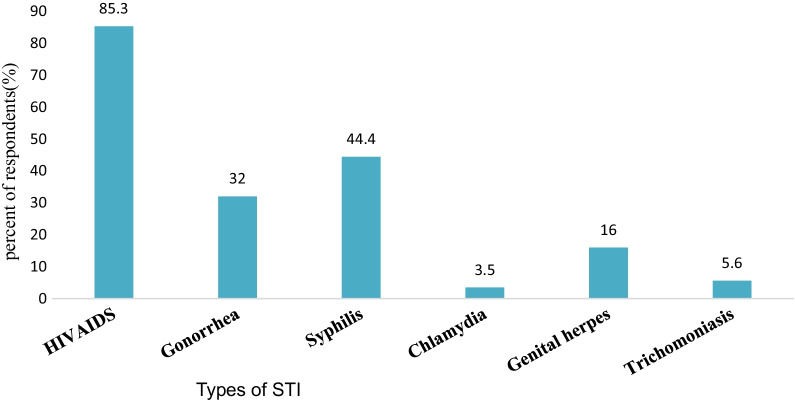
Types of STI-specific information sought by students in the University of Gondar (n = 462)

From the whole STI information seekers, the majority 394 (85.3%) of the respondents had sought about HIV/AIDS, 205 (44.4%) about Syphilis, 148 (32%) about Gonorrhea, 74 (16.0%) about Genital herpes, and 26 (5.6%) about Trichomonas’s (Fig. 4).

### Trust in STI information sources

The result of this study showed that about 257 (55.6%), and 238 (51.5%) of the study participants had a lot of trust in STI information gained from Health care providers and books respectively. Others like the internet, a family of friends, and televisions are also highly trusted STI information sources by university students. Of the total STI information seekers, 148 (32%) of them had never trusted STI information from Magazines (Table 5).

**Table 5:** Student’s Trust on Information Sources about STI at the University of Gondar (n = 462) 2021

STI information sources	A Lot (%)	Some (%)	A Little (%)	Not At All (%)
Health care provider	257 (55.6)	107 (23.2)	59(12.8)	39 (8.4)
Family of friends	159 (34.4)	187 (40.5)	84 (18.2)	32 (6.9)
Internet	208 (45.0)	136 (29.4)	79 (17.1)	39 (8.4)
Television	162 (35.1)	142 (30.7)	95 (20.6)	63 (13.6)
Radio	139 (30.1)	117 (25.3)	126 (27.3)	80 (17.3)
Magazines	66 (14.3)	108 (23.4)	140 (30.3)	148 (32.0)
Books	238 (51.5)	126 (27.3)	62 (13.4)	36 (7.8)

### Reason for Seeking Information on STI

The result of this study showed that about 338 (73.2%), 249 (53.9%), 185 (40.0%), 131(28.4%), 117 (25.3) of the respondents' primary reason was to seek STI information for STI prevention, To know STI sign &symptoms, about STI treatment, about STI diagnosis mechanisms, and global and local burden of STI respectively. Whereas about 103 (22.3) of them had never looked for information on the global and local burden of STI (Table 6).

**Table 6:** Reason for seeking information on STI among students at the University of Gondar (n = 462) 2021

Reasons	Primary reason (%)	Secondary reason (%)	Tertiary reason (%)	Never (%)
For STI prevention	338 (73.2)	90 (19.5)	24 (5.2)	10 (2.2)
To know STI sign &symptoms	249 (53.9)	151 (32.7)	33 (7.1)	29 (6.3)
About STI treatment	185 (40.0)	169 (36.6)	71 (15.4)	37 (8.0)
About STI diagnosis mechanisms	131 (28.4)	162 (35.1)	108 (23.4)	61 (13.2)
For global and local burden of STI	117 (25.3)	126 (27.3)	116 (25.1)	103 (22.3)

### Factors associated with STI information-seeking behavior

To select significant factors associated with STI information-seeking behavior, bi-variable and multivariable logistics regression were undertaken. The result in bi-variable logistic regression analysis indicated that students field of study, year of study, age, sex, voluntary club participation, internet access, digital literacy, sexual experiences, had STI symptoms, Perceived susceptibility, perceived severity of STI, and Family history were significantly associated with STI information-seeking. Variables with a p-value less than 0.2 were considered for multivariable analysis. In multivariable analysis field of study, year of study, internet access, digital literacy, sexual experiences, had STI symptoms, Perceived susceptibility, and perceived severity of STI were significant factors associated with STI information-seeking.

The odds of STI information-seeking among Health science undergraduate students is 2 times higher than non-health science students (AOR = 2.19, 95% CI = 1.16, 4.11). The findings of this study indicated that students whose year of study was fourth and fifth years were 4.7 times (AOR = 4.77, 95% CI = 2.75, 8.29) and 5 times (AOR = 5.45, 95% CI = 2.48, 12.01) were more likely to seek STI information when compared to second-year students respectively.

Participants with high digital literacy levels were 5.7 times (AOR = 5.75, 95% CI = 3.91, 8.47) more likely to seek STI information when compared with those who had low digital literacy levels. The other significant variable is internet use. The result indicated that the odds of seeking STI information among respondents who had Internet access were 2 times (AOR = 2.24, 95% CI = 1.27, 3.94) higher when compared to those who had limited access to the internet.

When compared with respondents who didn’t have a sexual history, participants who had sexual experience were 2 times more likely to seek STI information from different information sources (AOR = 2.33, 95% CI = 1.56, 3.48). In this study, respondents who had STI symptoms were 4 times more likely to seek STI information compared to respondents who didn’t show STI symptoms (AOR = 4.19, 95% CI = 2.14, 8.18). In addition to this, the participant who had high perceived severity of STI were 2 times more likely to seek STI information compared to participants who had low perceived severity of STI (AOR = 2.16, 95% CI = 1.45, 3.22). the participant who had high perceived susceptibility to STI were 5 times more likely to seek STI information compared to participants who had low perceived susceptibility to STI (AOR = 5.05, 95% CI = 3.29, 7.75). In multivariable analysis sex, age, voluntary club participation, and family history of STI were not significantly associated with STI information-seeking (Table 7).

**Table 7:** Factors associated with STI information-seeking among students

Variables	STI Information Seeking		COR (95%)	AOR (95%)
	Yes N (%)	No N (%)		
Field of study				
Health-related	72 (15.6)	24 (6.5)	2.66 (1.64,4.32)	2.19(1.16,4.11)*
Non-health-related	390 (84.4)	346 (93.5)	1	1
Year of study				
Second year	100 (21.6)	146 (39.5)	1	1
Third year	164 (35.5)	169 (45.7)	1.41 (1.02, 1.98)	1.36 (0.89.2.09)
Fourth year	136 (29.4)	41 (11.1)	4.84 (3.14, 7.46)	4.77 (2.75,8.29)*
Fifth year	62 (13.4)	14 (3.8)	6.47 (3.43,12.18)	5.45 (2.48, 12.01)*
Internet access				
Yes	426 (58.9)	297 (41.1)	2.91 (1.90,4.45)	2.24 (1.27, 3.94)*
No	36(33)	73 (67)	1	1
Digital literacy				
High	281 (60.8)	90 (24.3)	4.83 (3.57,6.53)	5.75 (3.91,8.47)*
Low	181 (39.2)	280 (75.7)	1	1
Sexual experience				
Yes	202 (43.7)	260(56.3)	2.38 (1.76,3.21)	2.33 (1.56,3.48)*
No	91 (24.6)	279 (75.4)	1	1
Had STI symptoms				
Yes	75 (16.2)	20(5.4)	3.39 (2.03,5.67)	4.19 (2.14,8.18)*
No	387 (83.8)	350(94.6)	1	1
Perceived susceptibility				
Concerned	250 (54.1)	57 (15.4)	6.48 (4.63,9.06)	5.05 (3.29,7.75)*
Not Concerned	212 (45.9)	313 (84.6)	1	1
Perceived severity				
Severe	253 (54.8)	82 (22.2)	4.25 (3.13,5.77)	2.16 (1.45,3.22)*
Not severe	209 (5.2)	288 (77.8)	1	1

*p < 0.05; 1 = reference

## Discussion

This study tried to discover out students' information-seeking behaviors about STI. The result from this study is used to pick out the feasible STI information sources preferred by the University students and factors associated with STI information-seeking. In addition to this, the findings will give instructions to the University students to use various STI information sources to prevent themselves from STI. The study result may be used by policymakers and different stakeholders to formulate a plan based on the findings to reduce the burden and transmission of STI.

Findings in this study indicated that 55.5% (CI = 52.3, 58.9) of the university students were sought STI information from different information sources in the past 12 months, which is consistent with a study conducted in Malaysia (57.1%) [25]. On the other hand, the result of this study is lower than a study conducted in Nigeria which suggested that Sexual and reproductive health information-seeking was 73.6% [5]. The possible reasons might be the former study were reported all sexual and reproductive health Information-seeking behavior while the current study focused on STI Information-seeking behavior. Similarly, this study is lower than another study done in Malaysia (62.4%) [15]. This variation might be due to the differences in IT infrastructure among those study areas because the majority of the respondents were sought STI information from the internet. The other possible explanation for this may be the variations in the awareness of students towards STI information sources.

Regarding STI information sources, around 56.9% (263) of STI information seekers preferred the internet as their primary STI information source, which is consistent with the previous study which stated that the internet was the main source for sexual and reproductive health information [26]. Students may have learned that STI information is crucial in preventing STI transmission by obtaining STI information on the internet, which may have improved their health. However, This finding is lower than a previous study conducted in Malaysia in which 84.5% of the respondents have preferred the Internet as their primary health information source [3]. Similarly, the result is lower than the previous study conducted on Ghanaian university students in which 67.7% of the respondents have preferred the Internet as a primary source for health information purposes [27]. This discrepancy could be explained by the fact that the prior study focused on general health Information seeking behavior, whereas the current study focuses on STI Information seeking behavior. Another reason for the disparities could be a lack of understanding among students about the relevance of STI information sources and a limited culture of using them for their health [28].

According to this study, health science students were more likely to seek STI information than non-health students. This could be because health science students are well enough in the transmission and preventive processes of sexually transmitted infections, and they seek health information for their school/college lessons. Another significant factor in this study is the year of study. Students whose year of study was fourth and fifth years were more likely to seek STI information than second-year students. This finding is consistent with a prior study, which found that people with lower educational levels were less likely to seek health information [29]. This could be because as a student's year of study increases, their understanding of the effects of sexually transmitted illnesses on their health grows, allowing them to seek STI information [30].

This study indicated that internet access was a significant factor associated with STI information-seeking. When compared to those with limited internet access, those with internet access were more likely to seek STI information. This result is consistent with a study conducted in Ghana [27]. Similarly, another study reported that Participants with access to STI information were considerably more likely to be aware of STI prevention mechanisms than those with limited access to STI information [15]. This result is also supported by a previous study conducted in Ethiopia in which respondents who had access to the internet were 1.97 times more likely to use EHIRs than those who did not [31]. The possible explanation for this could be that most students preferred the internet as a primary STI information source as discussed above in this study. This study has also found that STI information-seeking was associated with respondents’ digital literacy. That is respondents who had high digital literacy levels were 5.7 times more likely to seek STI information when compared with those who had low digital literacy levels. This finding is supported by a study conducted in Ethiopia which stated that When compared to those with low computer literacy, those with good computer literacy were 3.12 times more likely to use electronic health information resources to seek health information [31].

Sexual experience is another significant factor associated with STI information-seeking. This study indicated that students who had sexual experience were more likely to seek STI information when compared with those who didn’t have sexual experiences. This is supported by a previous study, which found that individuals in a relationship or dating status were more likely to seek STI information [15]. Similarly, respondents who had STI symptoms were more likely to seek STI information compared to respondents who didn’t show STI symptoms. This finding is consistent with a study conducted in Malaysia in which participants had previous experience of STI symptoms that were positively associated with STI information-seeking [15]. This could be due to respondents who had STI symptoms seeking STI information from online and offline sources to increase their awareness about prevention and diagnosis of STI. Another possible explanation is that respondents with STI symptoms sought STI information to obtain accurate information about STI symptoms to compare with their present symptoms, allowing them to seek additional treatment and reduce infection transmission.

This study showed that participants who had high perceived severity of STI were more likely to seek STI information than respondents who had low perceived severity of STI. This finding is consistent with a former study in which perceived severity of STI was the predictor variable for STI information-seeking [15] and one in Ethiopia [30]. If students feel worried by the infection, their assumption that STIs have a major influence on their health may place them to engage in STI prevention behavior. Students who think STI is a serious problem want to know about the risk factors, preventative strategies, diagnosis, and possible treatments [15]. In this study, Respondents who were concerned about contracting an STI were more likely to seek STI information than those who were not bothered at all. This result is consistent with a previous study conducted in Malaysia [3].This indicated that a person's perceived susceptibility to health problems had a favorable impact on their usage of the Internet to seek health information, resulting in a reduction in the prevalence of STI. Although this study provided evidence of Information-seeking behavior on sexually transmitted infections among university students, it should have had certain limitations. Self-administered data collection methods may be influenced by socially desirable replies, especially when premarital sex is considered unacceptable in Ethiopia, despite appropriate safeguards such as protecting their confidentiality and anonymity. As a result, data on sexual history may be under-reported. The study was a facility-based cross-sectional study, hence the causal inference between variables may not be articulated. There was a chance of recall bias in the study because it looked at STI information-seeking in the past year.

## Conclusion

In general, the proportion of STI information-seeking among University students was low. Respondents preferred the Internet as a primary STI information source followed by health care providers. Field of study being health, year of study, internet access, digital literacy, sexual experiences, had STI symptoms, perceived susceptibility, and perceived severity of STI were significant factors associated with STI information-seeking behavior. More studies are required to improve evidence-based practices on STIs prevention and control programs.

## Data Availability

The data set gathered and/or analyzed for the current study will be made accessible upon request of the corresponding author.

## Abbreviations

SRH: Sexual and reproductive health
STIs: Sexually Transmitted Infections
HIV: Human immunodeficiency virus
AIDS: Acquired immunodeficiency syndrome
WHO: World Health Organization
